# A new dynamic tactile display for reconfigurable braille: implementation and tests

**DOI:** 10.3389/fneng.2014.00006

**Published:** 2014-04-08

**Authors:** Paolo Motto Ros, Vittorio Dante, Luca Mesin, Erminio Petetti, Paolo Del Giudice, Eros Pasero

**Affiliations:** ^1^Center for Space Human Robotic, Istituto Italiano di Tecnologia (IIT@PoliTO), with Dipartimento di Elettronica e Telecomunicazioni of Politecnico di Torino, and with Istituto Nazionale Fisica Nuclearesezione di Torino, Torino, Italy; ^2^Istituto Superiore di Sanità di Roma and with Istituto Nazionale Fisica NucleareSezione di Roma, Italy; ^3^Dipartimento di Elettronica e Telecomunicazioni of Politecnico di TorinoTorino, Italy; ^4^Dipartimento di Elettronica e Telecomunicazioni of Politecnico di Torino and with Istituto Nazionale Fisica NucleareSezione di Torino, Torino, Italy

**Keywords:** assistive technology, user interface human factors, system analysis and design, tactile displays, braille reading aids

## Abstract

Different tactile interfaces have been proposed to represent either text (braille) or, in a few cases, tactile large-area screens as replacements for visual displays. None of the implementations so far can be customized to match users' preferences, perceptual differences and skills. Optimal choices in these respects are still debated; we approach a solution by designing a flexible device allowing the user to choose key parameters of tactile transduction. We present here a new dynamic tactile display, a 8 × 8 matrix of plastic pins based on well-established and reliable piezoelectric technology to offer high resolution (pin gap 0.7mm) as well as tunable strength of the pins displacement, and refresh rate up to 50s^−1^. It can reproduce arbitrary patterns, allowing it to serve the dual purpose of providing, depending on contingent user needs, tactile rendering of non-character information, and reconfigurable braille rendering. Given the relevance of the latter functionality for the expected average user, we considered testing braille encoding by volunteers a benchmark of primary importance. Tests were performed to assess the acceptance and usability with minimal training, and to check whether the offered flexibility was indeed perceived by the subject as an added value compared to conventional braille devices. Different mappings between braille dots and actual tactile pins were implemented to match user needs. Performances of eight experienced braille readers were defined as the fraction of correct identifications of rendered content. Different information contents were tested (median performance on random strings, words, sentences identification was about 75%, 85%, 98%, respectively, with a significant increase, *p* < 0.01), obtaining statistically significant improvements in performance during the tests (*p* < 0.05). Experimental results, together with qualitative ratings provided by the subjects, show a good acceptance and the effectiveness of the proposed solution.

## 1. Introduction

The braille system is one of the most important ways to access information by visually impaired people. As reported by National Braille Press (2011) (NBP), blind people have difficulties to find a job (with a lost productivity in the United States of $8.0 billion per year), but the majority of those employed are braille readers.

Even if braille might be in competition with speech synthesis, it offers the same natural approach to reading as for sighted people (García, 2004; Shimomura et al., 2010). Interviewing participants to our experiments about the effectiveness/usefulness of speech synthesis interfaces rather than of tactile ones, a strong interest in the latter has emerged, given that they offer a superior control on the information flow. Even if braille is more difficult to grasp, in the end it guarantees the needed opportunity to interact with the information source and it enables the user to have an active approach in retrieving the desired information. Conversely, with speech synthesis the user tends to have a passive role, with the major drawback of requiring an increased effort to focus attention as needed and to retain the useful part of the information flow. This is why, despite its longevity and the technical difficulties to equip an existing information system with braille (it requires custom hardware, unlike speech synthesis), it is still an important means to enhance the quality of blind people's life.

The first braille solutions were embossed books, but they are bulky, expensive, and, most notably, with a fixed content. Then braille printers and bars were introduced, which can be interfaced with common information systems thus allowing visually impaired people to access digital contents. However, the former still rely on papers as a reading support, and the latter are strictly limited to standard braille. Despite its widespread adoption, braille is not the only embossed technique developed to provide tactile information to blind people: alternatives are the jumbo braille, Fishburne, ELIA or Moon code, besides raised print or tactile graphics (Cryer et al., 2008), but none of them has received enough attention to become a real competitor (Cryer et al., 2009). It should be noted, however, that jumbo braille is just a “revision” of the standard braille, having the same encoding technique but with enlarged cell spacing and dot size. Another interesting braille-based approach makes use of a common mobile device used for implementing a new method for presenting braille characters (Rantala et al., 2009). Furthermore, raised print and tactile graphics do not involve information encoding in a strict sense, since they just reproduce a visual information into a tactile experience: it is the only feasible approach whenever there is not an appropriate translation by other means, such as for drawings, icons or similar. We can say that an ideal tactile interface should combine the ability to provide both textual and graphical information within the same device in a seamless way.

Nowadays the trend in this field is gearing toward dynamic graphical tactile displays: the purpose could be either to provide a kind of tactile graphical user interface (Schiewe et al., 2009) (since actually almost all the ICT systems used in daily life have a graphical user interface) or to go beyond standard text by introducing tactile icons (Pietrzak et al., 2009). For these reasons new tactile interfaces have to be devised, able to overcome the main limitation of commonly available braille bars strictly focused on representing text encoded in standard braille. They should be able to provide arbitrary graphical information, but still with the major requirement of guaranteeing a standard and familiar reading approach. Given this possibility, it is interesting to take the opportunity to make the interface deal with different braille variations, in a similar way to what was studied for a printing system (Hara et al., 2002); this would be beneficial for new learners (especially late-blind persons) and people with a poorer tactile acuity (Cryer et al., 2009), or just to make the user more comfortable.

The design of tactile interfaces requires first to understand physiological, psychophysical and neurological aspects; the sense of touch relies on four different kinds of mechanoreceptors (excluding hair follicles) (Johnson, 2001; Hale and Stanney, 2004), each of them responding to a particular type of mechanical stimulus (usually through a pin pressing the fingertip). Merkel cells (also known as SA1, Slowly Adapting type 1) have a high spatial resolution (discrimination begins at a resolution of 0.5mm, but the response becomes significant with a spatial frequency of 1mm^−1^) and a wide dynamic range, while Meissner cells (also known as RA, Rapidly Adapting) have a great sensitivity and a poor spatial resolution (in the range 3–5mm) (Johnson, 2001). Another important distinction between them is that they respond to static or dynamic stimulations, respectively. These physiological properties of the tactile response influenced the development of tactile devices, which follow two possible approaches: the first relies on indentation (or displacement), where the displacement carries the information^1^; the second is based on a vibrotactile mode, where the information is encoded in the frequency of the stimulation. The second one seems to be more widespread, possibly due to the simpler engineering of compact actuators, since it is not needed to hold the pin pressing the fingertip and the minimum required displacement is low, about 100μm (Yoon and Yu, 2008). The main drawback is the phenomenon called *adaptation*, by which, after a first period of correct perception, the fingertip becomes substantially insensitive (Way and Barner, 1997); adaptation is a common issue across all the sensory modalities, but among all the mechanoreceptors, RA are the most prone to adaptation (Kaczmarek et al., 1991). On the other hand, an indentation approach provides a more natural and comfortable feeling, similar to common embossed surfaces, and this is why it is the most used method for braille bars.

Besides the aspects outlined above, the issue of how the tactile acuity relates to aging has to be taken into account, given that the average age among blind people is higher than that of sighted people (because of late-blind persons). According to Lighthouse International (2012), among people who are blind worldwide, 58% are older than 60, with the first three causes (cataract, glaucoma and macular degeneration) all age-related. Assessing the tactile acuity decline with age is a difficult task, to the best of our knowledge still being a debated issue. It has been both reported a tactile acuity decrease by sighted and blind (by approximately the same rate) (Stevens et al., 1996), and no observed decrease in blind people (Legge et al., 2008). This latter result was explained with the difference between *active* and *passive* touch, the first one including kinesthetic signals from motor movements of the hands or fingers, besides bottom-up tactile stimulation by the device. Another proposed explanation is the continuous practice of tactile ability by blind people, but we can not expect people losing their sight in adult life to have the same ability. All of these analyses suggested us that the design of a flexible device, allowing the user to choose several aspects of the tactile rendering, might offer an option for age-dependent adaptation (as well as adaptation to other individual characteristics).

However, the relationship between the tactile acuity and the braille reading performance, with respect to aging, is difficult to assess, given that it could be heavily influenced by cognitive factors (Legge et al., 2008; Kalisch et al., 2012). Even if a negative trend was observed in the reading rate with respect to the decrease of the tactile acuity, no final conclusion can be drawn (Stevens et al., 1996). In any case, it seems clear that such an issue is highly dependent on each individual, thereby suggesting that it would be a welcome improvement to allow users to choose themselves the size and spacing of tactile information to be reproduced.

In this paper the design, development and test of an innovative dynamic graphical display are discussed. Piezoelectric actuators provide static stimulations; the content can be refreshed at a high rate. Therefore “static” and “dynamic” here are not in conflict: the first refers to the static stimulation (opposed to vibrotactile approach), and the second to the capability of quickly update the rendered information.

Such a device is part of a larger project aiming to study and design a novel aid, portable and autonomous, to allow blind people to access both graphical and textual information. They can not be regarded as disjoint issues, given that often they are intermixed on the same page the user is trying to read. So the user should be able to interact with the device and the information content in a seamless way, switching between these two main modes in real-time. Furthermore, in our experience, the possibility to customize every aspect of the tactile representation is highly welcome, because of particular needs or just because of individual preferences. In order to reach this challenging goal, it is clear that a display with a higher resolution than standard braille is needed. The proposed device is studied and designed to be used just like a PC mouse. On the bottom side, it has a camera devoted to acquire printed information, either characters (to be encoded in braille) or graphics, while on the top side there is the tactile display which is the main focus of the present paper. The user moves the device on the paper and in real-time the elaborated information “seen” by the device are rendered on the tactile display. The user experience is similar to the proposal presented in Saranyaraj et al. (2011), but not limited to braille.

Regarding textual applications, a software has been developed (Motto Ros et al., 2009) to recognize single characters, assemble them in spell-checked words which can be either rendered in tactile patterns or through speech synthesis (not covered in the present work). The user is informed in real time (in either of the above modes) about the alignment of the device with respect to the underlying text. This helps the user to read printed information in a highly interactive way, either following the text or just exploring the paper to search for the desired part.

Regarding graphical applications, taking advantage of the flexibility of the tactile display, in Motto Ros and Pasero (2011) it has been interfaced with a digital vision sensor (working in a similar way as the retina does) (Lichtsteiner et al., 2008). The whole application has a neuromorphic approach, which can be considered as a kind of event-based way of processing data: in this case, visual events (local temporal intensity changes) are translated into tactile stimulations.

In section 2, we describe the proposed device and the experimental protocol designed to validate it; results of the experiments are shown in section 3; an evaluation of our device and future perspectives are provided in section 4; finally, Appendix A provides some details on the subjects participating in experimental tests together with additional results.

## 2. Materials and methods

**Figure 1:**
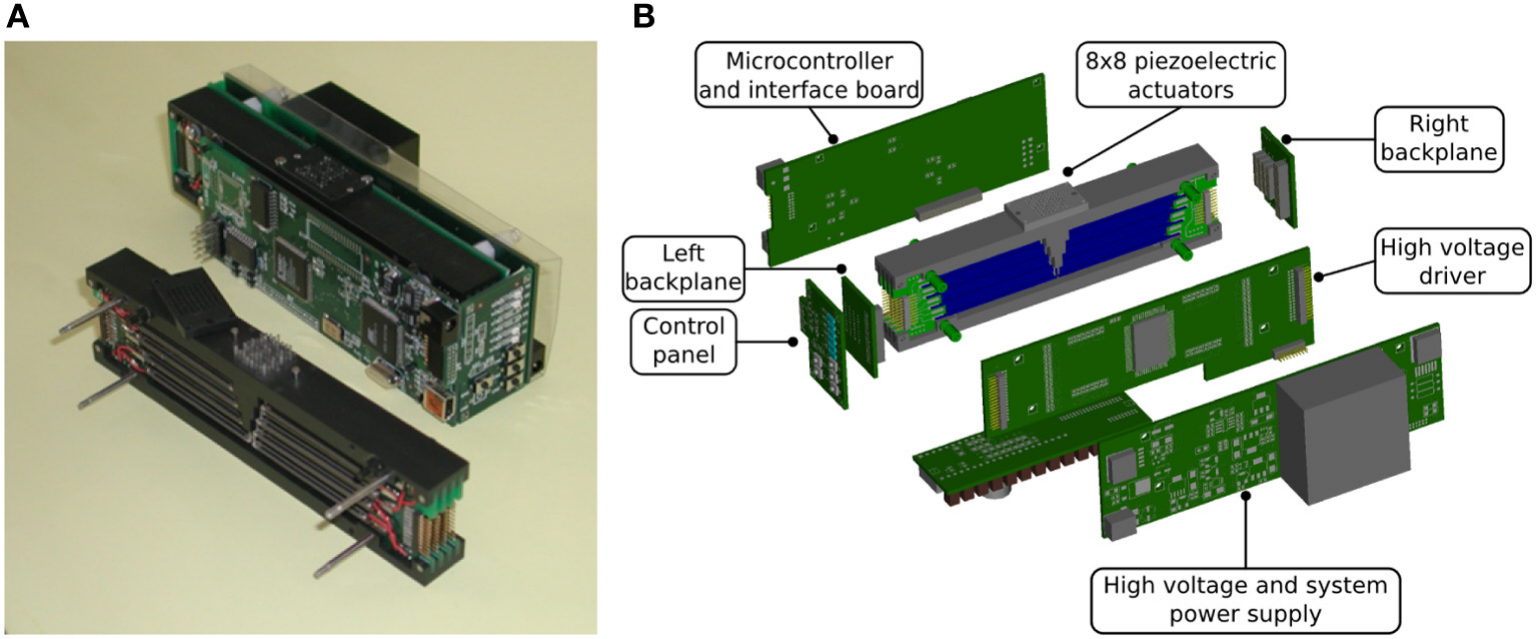
**Overview of the proposed device**. **(A)** View of the tactile display (with and without the control boards), **(B)** exploded view drawing of the tactile display.

The proposed tactile display is designed around a core mechanical structure, with cantilevers for the bimorphous piezoelectric reeds and holding tactile pins in the correct position; all around this part, PCBs with the needed control electronics are placed. Such an arrangement (shown in Figure 1) allows the minimization of the overall size of the device.

**Figure 2:**
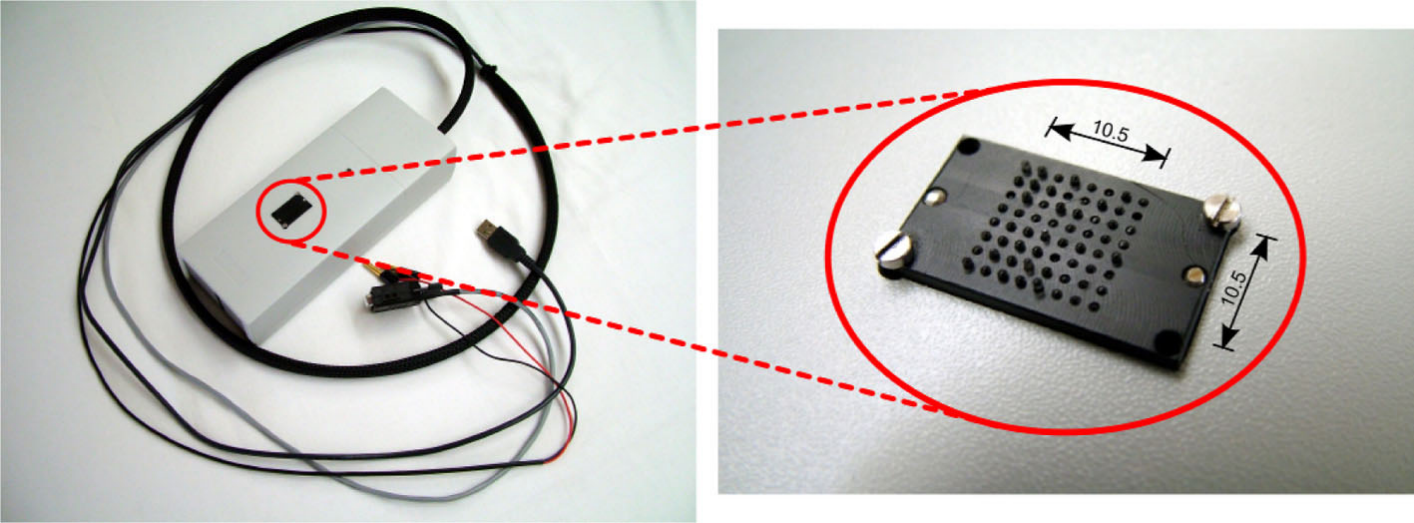
**Overview of the tactile display**. Packaged view on the **left**, close up view of the tactile display (during a protocol functional test) on the **right**; dimensions are in mm.

**Figure 3:**
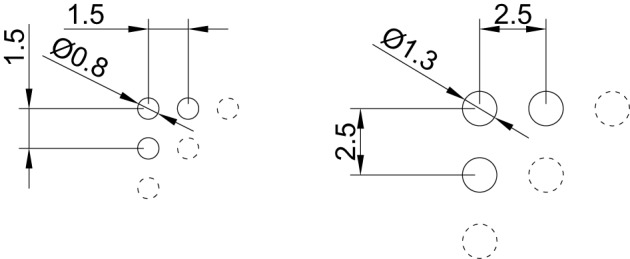
**Pin arrangement comparison between the proposed solution (left) and standard braille (right); dimensions are in mm**.

The top side view of the tactile matrix is shown in Figure 2; pins are made of plastic rods with a rounded ending. The diameter of each pin is 0.8mm, the gap is 0.7mm, resulting in a interaxial distance of 1.5mm; the matrix size is 8 × 8. Figure 3 compares the resulting arrangement with the standard braille. These design choices were made to better match the minimum spatial resolution recognized by Merkel cells (SA1) mechanoreceptors, whose response begins to be noticeable/significant with a resolution of 0.5mm/1mm, respectively (Johnson, 2001), even if it is commonly reported a two-point limen of 1mm as the practical useful resolution (Hafez, 2007).

**Figure 4:**
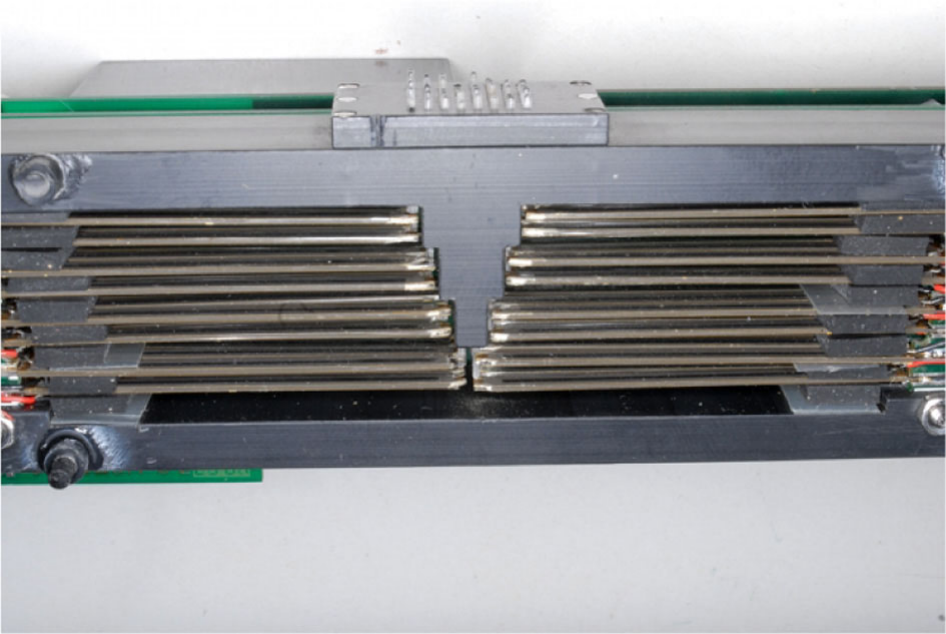
**View of the arrangement of piezoelectric elements into layers and sublayers**.

**Figure 5:**
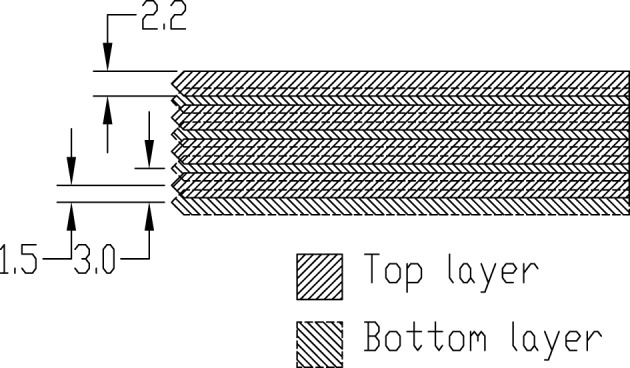
**Top-view drawing of a single actuators layer; dimensions are in mm**.

Pins are pushed up by piezoelectric actuators from Murata (part number PKF02C5, muRata, 2003); to lower the pins we rely on the pressure applied by the fingertip, as done in common braille devices. Pins are not mechanically tied to the reeds, but simply leaned against them, not to have a hinge which would be subject to mechanical stress. Since the width of each reed is 2.2mm, it is not possible to build a single layer of actuators for each tactile row. So, they have been arranged in layers and sublayers: four actuators have been used on each upper sublayer to move the odd columns, other four on each lower sublayer for the even columns. Then each layer is progressively displaced to put in contact with the corresponding row of pins (with the first layer related to the first row). In order to minimize the height of the overall device, half of the actuators are placed on one side of the tactile matrix, the other half on the opposite side. Figures 4, 5 show the arrangement: for each side there are four layers, for each layer two sublayers, and for each sublayer four reeds; thus in total there are all the 2 × 4 × 2 × 4 = 64 actuators. In order to maximize the full range of displacement (in both directions) allowed by actuators, their resting position does not correspond to the lowest position of the tactile pins: indeed a negative voltage is used to bend the reeds down thus taking and holding the pins down; on the opposite, a positive voltage will raise the pins making them perceivable by the user. When raised, the resulting dot height is 1mm.

**Figure 6:**
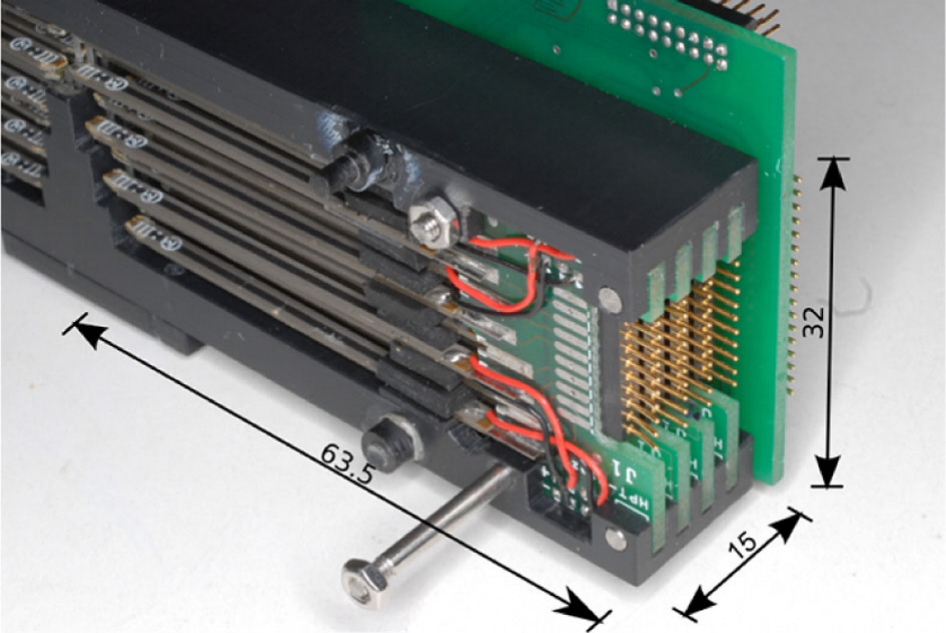
**View of the backplanes holding the piezolectric element, arranged in columns; dimensions are in mm**.

From the electrical standpoint, the piezoelectric reeds are grouped by columns. In total, there are four columns with eight actuators each; each column is interconnected with a PCB (see Figure 6) which in turn is plugged onto a backplane (one per side). All piezoelectric actuators are driven by a Supertex HV507 driver (Supertex Inc, 2008), which provides in one package all the needed interface electronics (CMOS compatible inputs, 64 high voltage push-pull outputs). The high voltage is supplied by the DC-DC converter Deutronic DH12-0,5K (DH12/DH24, 2006); the actual high voltage applied to actuators can be digitally controlled through a DAC. Actual driving voltage is 200V, which corresponds to an applied average force of 83.3mN (muRata, 2003), ensuring an effective stimulation (Hale and Stanney, 2004); this setup was optimized in preliminary tests with visually impaired people.

The control electronics is made of a Xilinx Spartan XCS30XL FPGA (Xilinx, 2008) and an Atmel 89C5132 MCU (8051 family) (Atmel, 2007). This design choice was considered for possible future applications, which could require integrating and customizing the tactile display in new appliances. In the current version, the FPGA is used as a kind of tactile frame-buffer, much like the video memory is used in common electronic devices: it implements a dual-port RAM, with one port accessed by the MCU and the other one used to scan the content and refresh the tactile matrix. The MCU implements the protocol used to exchange data with ICT systems, writes the incoming data to the frame-buffer and, with the integrated DAC, controls the DC-DC converter. Currently the system is equipped with both a USB and a RS232 serial interface; for the purpose of backward software compatibility, we implemented a COM emulation mode for the USB. We also implemented a Bluetooth chipset, as an alternative to the USB, to make the device independent of physical connection with a PC, or to interface it to compliant devices of any kind (e.g., mobiles, ebook readers). Although this feature is implemented and working, it was not used in the present context. The involved protocol is really simple, since each eight-byte packet includes a command, options, data and a checksum (respectively one, one, five, and one byte). To fully setup the desired content on the display, two packets are needed (one per upper/lower half of the matrix), each one carrying one byte per row (one bit per up/down pin position) as data. Alternatively, for configuration purposes, the packet can code for tunable parameters such as the refresh frame rate or the force exerted by the actuator (through the voltage applied to the piezoelectric cells). The current baud rate is set to 9600, so in about 10 ms the information can be transferred, corresponding to a 100frames/s refresh rate^2^, higher than similar interfaces (Vidal-Verdu and Hafez, 2007).

In the current implementation, most power consumption is due to the high-voltage module (maximum current less than 100mA, worst case). On top of this, dynamic variations are negligible, due to the fast transitions between the up and down pin positions. The above base power consumption can be lowered by making it use-dependent; a custom high-voltage module has been designed and tested for this purpose, but not implemented in the prototype used for the tests reported here. Power consumption due to the MCU and FPGA are obviously widely varying in time, with peak current around 200mA over 2–3ms (during packet transmission). Overall, given the above estimates, the device could be powered by Li-Pol or Li-ion batteries (two cells), thus making the device portable.

### 2.1. Experimental protocol

Experiments with experienced braille readers were performed to test the readability of textual information reproduced by our device. The subjects could choose between three presentation modalities and the optimal pace of reading. Then, two different velocities of presentation of characters (the optimal and a faster one) were tested. Moreover, strings with different information content were considered. Some tests were performed in random order to check for possible effects of training in improving performance. Finally, a questionnaire was proposed to ask for some qualitative considerations.

#### 2.1.1. Participants

Eight volunteers (five females and three males) participated in the experiment (average age 50 years, range 15–70). Seven participants were blind and one had a visual impairment due to retinal macular degeneration. Six subjects were impaired from birth, two of them became impaired at age 2 and 25, respectively. All participants had no additional impairments. The participants were experienced braille readers (they started studying braille at 6 years old, with the exception of the subject who became blind at age 25, who started then studying braille; the average number of years reading braille was 41, range 9–63); all of them are mother-tongue in Italian. Moreover, they had experience as braille writers or note takers. Additional participants' information is reported in Appendix A.

#### 2.1.2. Technical settings

The reading rate can be determined in two ways: self-paced or fixed by the control application. The first option can be implemented using PC keyboard or mouse, but the measured response times could be not accurate as needed, since their order of magnitude is of 10 ms (up to 100 or above) (Shimizu, 2002; Plant et al., 2003) and hence not suited for our purposes. These issues can be overcome by developing custom measurement devices like RTbox (Li et al., 2010), but, since we are interested in the *average* optimal reading speed and not in the maximum one, we preferred to let the users find their optimal rate (on a trial and error basis, with the experimenter tuning the speed) and then to maintain it fixed. The details of timings are as follows: at time *t*_*i*_ the *i*th character of the test sequence is rendered on the tactile matrix; at *t*_*i*_ + *T*_*p*_ the display is cleared; the blank period lasts until *t*_*i*_ + *T*_*p*_ + *T*_*b*_ when the next (*i* + 1)th character is made available (*t*_*i* + 1_ = *t*_*i*_ + *T*_*p*_ + *T*_*b*_). Thus *T*_*p*_ is the persistence interval time of the character on the display (chosen on a per user basis, according to her/his preference) and *T*_*b*_ defines the blank period. In our experiments *T*_*p*_ = *T*_*b*_, so that the rate can be unequivocally defined by *T*_*p*_.

By “optimal rate” we mean the rate at which users feel more confident (it does not mean that it is the rate for which no errors are made, see section 2.1.3). Furthermore, another 33% faster rate was tested in order to investigate the effect of reading velocity on the recognition accuracy. Thereafter different tests, with both optimal and faster rate, were performed to assess the reading accuracy.

The tactile display was placed on a table top during the experiment. The participants were instructed to hold their dominant hand (right hand for all subjects) on the pin matrix. Instructions were verbally provided by the experimenters during the experiment. The tactile display was controlled by an application written in Python programming language, working as a kind of sequencer of braille symbols. Since we did not need a full featured framework, e.g., like PsychoPy (Peirce et al., 2011) or DMDX (Forster and Forster, 2003), we developed an *ad hoc* multi-threaded application with a cooperative scheduler which accurately tracks execution timings.

**Figure 7:**
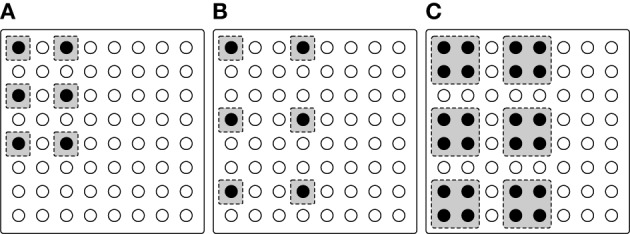
**Tested braille configurations**. **(A)** Standard layout. **(B)** Spaced layout, with an increased distance between pins. **(C)** Large layout, with four pins making one braille dot.

As stated above, characters were presented for a time period followed by a blank interval (same duration), one at a time. The six-dot braille symbols used in the experiment were lowercase letters of the English alphabet. Three presentation modalities of braille characters were proposed (shown in Figure 7), exploiting the high density pin matrix:

1. *Standard braille* (Figure 7A) is intended to resemble as much as possible (given the display geometry) the braille standard (Dixon, 2010), with a single pin (diameter 0.8mm) as braille dot and one row/column left blank between two adjacent dots (spacing 2.2mm).
2. *Spaced braille* (Figure 7B), same as above, except that the spacing is nearly doubled (i.e., two rows/columns left blank, spacing 3.7mm): the idea is to make dots more distinct, especially for elderly people whose tactile perception ability can be poor.
3. *Large braille* (Figure 7C), where each dot is represented by a 2 × 2 array of pins. Since the spacing between two adjacent pins (0.7mm) is less than the two-point limen of 1mm (Hafez, 2007), these square dots should be perceived as a single braille dot with an edge of 2.3mm. Pin spacing is the same as in the Standard braille configuration (2.2mm).

#### 2.1.3. Procedure

The subject was first invited to explore the dimensions of the device and its pin matrix with the hands, in order to familiarize with it. Meanwhile, the experimenter described the device and the experiment for about 5 min.

Then, the whole English alphabet was provided in sequential strings of three characters, with each of the three presentation modalities at a low rate, so that the subject could choose the preferred modality. Six subjects chose modality 1, one subject modality 2, and another preferred modality 3. Then, strings of three randomly chosen characters were provided with different rates, decreasing the duration till the subject could recognize about three to four characters out of five. Such a duration was considered as the optimal one (low presentation rate) and a duration 33% shorter was used for the high rate presentation.

Then, the experiment started. It consisted in three sessions.

- In the first one, 12 strings of randomly chosen triplets of letters were provided to the subject, first with the low-rate presentation and then with the high rate. Letter triplets were chosen after a few trial and error pilot experiments: in particular, three was the maximum number of letters that could be effortlessly memorized by the subject, who had to be mainly focused on recognizing the characters. The task was to recognize single braille characters and the performance was defined as the fraction of correctly identified characters ($P=\frac{\text{Correct characters}}{\text{Total}}\times 100\%$).
- The second session consisted in presenting Italian words. Four sets of 12 words each were considered: two sets with short words of about three letters (median 3, minimum 2, maximum 5), to be presented at low or high rate; the other two sets were words of about five letters (median 5, minimum 3, maximum 6), again to be presented at different rates. Each subject worked on sets chosen in a different order. The task was to recognize the single word. The performance of the subject was defined as the fraction of correctly identified words ($P=\frac{\text{Correct words}}{\text{Total}}\times 100\%$).
- In the third session we used two sets of words ordered such as to form meaningful sentences (four to six words each, words with the same statistics as in the second session). Two sentences were proposed at low rate (first set) and then other two sentences were presented at high rate (second set). The performance was defined as the fraction of correctly identified words, as in the second session.

After completing all three sessions, the participants were asked to rate their experience in using the device considering subjective scales ranging from 1 to 10. Subjective evaluations were divided into three different groups: general properties, expected potentialities of the device and interest in having the device. The general properties of the device were evaluated by the following metrics: easiness, speed, accuracy (in reproducing characters), pleasantness, efficiency, versatility and general evaluation. Potentialities were ranked with respect to: teaching braille, teaching shapes (as the number of pins and their density allows to reproduce different shapes in addition to braille characters), providing tactile information substituting vocal synthesis for blind people with reduced hearing ability. Finally, subjects provided free comments on the device and suggestions for future developments. Collected results are shown in Table 1; further results, concerning the influence of various characteristics of the subjects on the performance, are reported in Appendix A.

**Table 1:** **Participants' reading results: subject identifier, order of experiments, performance in reading strings at low rate, strings at high rate, short words at low rate, short words at high rate, long words at low rate, long words at high rate, sentences at low rate, sentences at high rate**.

**Subject**	**Exp. order**	**Performance**							
		**Strings**		**Short words**		**Long words**		**Sentences**	
		**Slow (%)**	**Fast (%)**	**Slow (%)**	**Fast (%)**	**Slow (%)**	**Fast (%)**	**Slow (%)**	**Fast (%)**
1	12345678	83	66	92	92	77	85	100	93
2	12456378	70	56	77	85	85	92	81	100
3	12563478	79	27	77	85	85	54	91	33
4	12635478	85	36	77	92	100	29	100	100
5	12346578	89	82	100	85	85	92	100	93
6	12436578	93	80	85	77	92	77	100	100
7	12543678	97	73	100	100	85	92	100	100
8	12654378	61	61	100	54	77	54	100	100

For the sake of the table clarity, “low/high rate” has been replaced by “slow/fast.”

### 2.2. Data analysis

Non-parametric statistics was computed to test for significant relations. In a preliminary test, Friedman Two-Way analysis of variance (ANOVA) for repeated measures was performed to test if there were significant effects on the performance determined by the two following factors: information content (where increasing information content was assumed when considering random strings, random words and sentences) and reading speed (considering the two frequencies of presentation of characters). As Friedman analysis does not allow to test for possible interactions between the two factors, a classical parametric Two-Way ANOVA was also performed.

Then, we focused on testing the effect of specific single factors, after pooling data. Specifically, we were interested in testing the effect on reading performance of the following factors: information content contained in the data, training during the experiment (considering the experiments on words reading in the order in which they were performed by the subjects) and reading speed.

Specifically, Kruskal-Wallis non-parametric One-Way ANOVA was first performed to investigate possible significant effects on performance, neglecting dependences between groups (which were present, as the same subjects were tested on different tests). *Post hoc* pairwise comparisons were based on average group ranks. Then, in order to include group dependence, the performance of each subject was considered separately. When investigating the effect of information content, subjects' performance was computed as follows: on random strings by averaging between low and high rate, on words by averaging between short/long words and low/high rate, and on sentences by averaging between low and high rate. Slopes of the regression lines were considered. Wilcoxon signed rank test was performed to check whether the slopes were significantly positive.

To investigate the effect of training on improving the performance on reading words, data of each subject were considered in the order in which the tests were performed. The four results (corresponding to low/high representation rate of short/long words) were interpolated with a straight line. Wilcoxon signed rank test was then performed to check if the slopes were significantly positive.

Finally, to test the effect of reading speed, a two-sided Wilcoxon signed rank test was applied to investigate if the performance at low rate was significantly higher than those at high rate.

Possible correlations between years of practice in braille reading and average performance, and between character optimal duration and reading speed conventional braille were also estimated. Moreover, mean performance was studied in relation to the education level (ordered giving value 1 to basic, 2 to high school, and 3 to college graduated education) and occupation (ordered giving value 1 to people who were never employed, 2 to retired, and 3 to subjects who were currently employed). Non-parametric Spearman's rank correlation coefficient was computed and significance was tested using the permutation distribution (i.e., computing the probability that the correlation coefficient would be greater than or equal to the obtained one, by using a permutation test, in which correlation coefficients are computed after re-arranging data by all possible permutations).

## 3. Results

### 3.1. Character recognition performance

**Figure 8:**
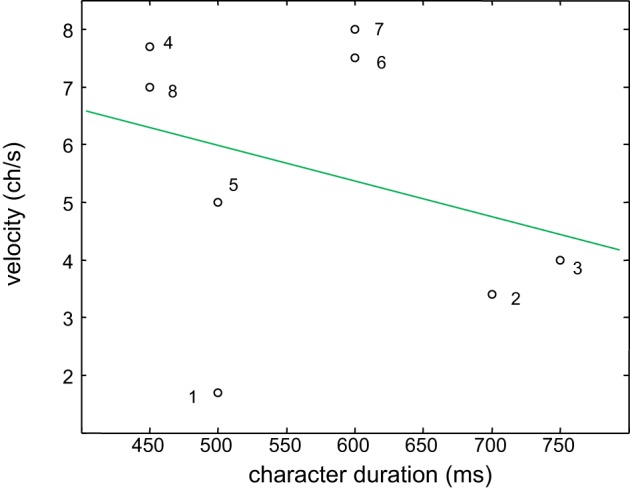
**Correlation between the choice of preferred persistence of the character reproduced by our tactile display and the self estimated velocity of reading of standard braille text**.

Figure 8 shows the relation between the choice of optimal duration of the character reproduced by our tactile display and the self estimated velocity of reading standard braille text. An attempt to fit data with a linear relation gave statistically poor results (Spearman coefficient *R* = −0.28, not significant, *p* = 0.5). Nevertheless, the negative trend is in line with the expected negative correlation between velocity of reading standard braille and time needed to recognize the character reproduced by our tactile display.

**Figure 9:**
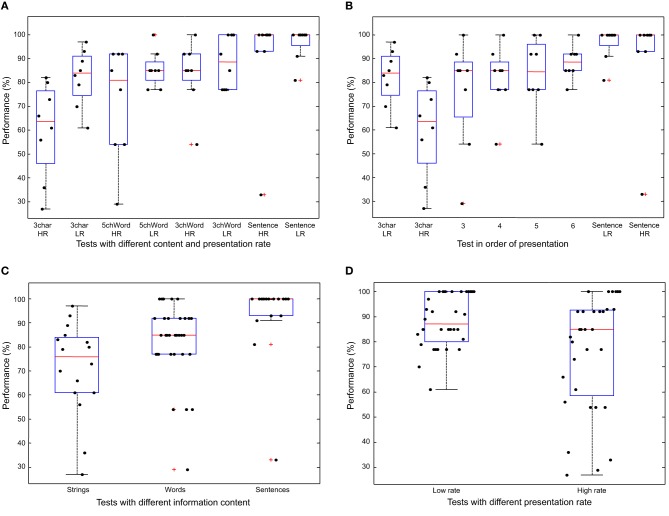
**Performances in recognizing characters, shown in box and whisker plots, indicating median, first and third quartiles, maximum and minimum values**. Outliers are shown individually. **(A)** Performances for different content and presentation rate: strings of three characters in random order (3char), words of average length of three and five characters (3chWords and 5chWords, respectively), and sentences, with low or high presentation rate of characters (LR and HR, respectively). **(B)** Performance as a function of the order in which tests were presented (the four tests on words were presented in random order to different subjects). **(C)** Performance as a function of the information content. **(D)** Performance as a function of presentation rate of characters.

Performance on specific tests are considered in Figure 9. The rationale for the experiments was the investigation of possible relations with performance of the information content to be retrieved, of training and of speed of the communication. Friedman Two-Way ANOVA indicated significant effects of the information content (*p* = 0.037) and of the reading speed (*p* = 0.0001) on performance. Classical Two-Way ANOVA confirmed these results and indicated that interactions between the two factors were not significant. Figure 9 shows the recognition accuracy in box and whisker plots, indicating median, first and third inter-quartiles, maximal and minimum values. Outliers are indicated individually. Moreover, individual data are shown.

Figure 9A shows the performance for different lengths of the character strings/words, for both high and low presentation rates.

Figure 9B shows the results in relation to the order in which the different tests were performed. The third to sixth tests were proposed in random order. Possible training effects were tested considering the performance in these four groups of data. Kruskal-Wallis test indicated that there is a statistically significant effect of training, and pairwise comparisons indicated that the third and the sixth experiments were statistically different, even neglecting that data were obtained by repeated measures on the same subjects. When introducing such an information, two-sided Wilcoxon signed rank test rejected the null hypothesis that the slope of performance with respect to subsequent experiments has zero median, indicating a significant effect of training in increasing the performance during the experiment (*p* = 0.015).

Figure 9C shows the results as a function of the information content, which increases going from random strings to words and sentences. Kruskal-Wallis test indicated that there is a statistically significant effect of the information content, and pairwise comparisons indicated that the results obtained working with sentences are statistically different from those obtained working with strings or words. No statistically significant effect was found when comparing results obtained reading strings or words. These results were obtained neglecting that data were obtained by repeated measures on the same subjects. Then, this information was included and Wilcoxon signed rank test was used to check that the slope of performance of the subjects with respect to experiments with increasing information content has a positive median. The test was highly significant, as all slopes were positive (*p* = 0.008). These results could be affected by the training effect. Nevertheless, a specific further contribution of the information content with respect to training is suspected, as the average slopes of performances with respect to the information content was higher than that of performance with respect to training.

Figure 9D shows the results with respect to the presentation rate of characters. Kruskal-Wallis test indicated no significant effect, when neglecting that the measurements were repeated on the same subjects. On the other hand, when considering paired comparisons studying the sign of the difference between the performance of equivalent tests done by the same subject at low and high rate, Wilcoxon signed rank test indicated that performance was significantly higher when the presentation rate was lower (*p* = 0.012).

### 3.2. Subjective ratings

**Figure 10:**
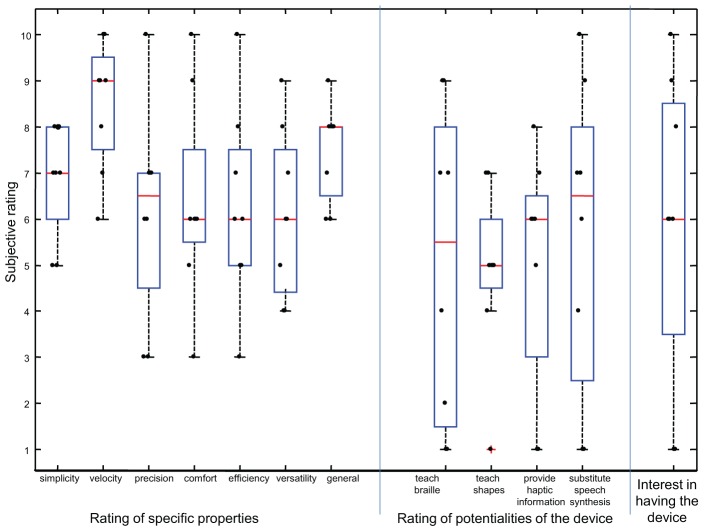
**Subjective ratings of properties of the prototype and expected potentialities, together with an indication of the interest of having a device similar to our tactile display**.

Subjective ratings are shown in Figure 10 in the form of box and whisker plots, indicating median, first and third inter-quartiles, maximal and minimum values. Individual ratings and outliers are shown. The ratings have a large spread, reflecting differences in perception of different users. Specific properties of the device (easiness, speed, accuracy, pleasantness, efficiency, versatility) obtained high ratings, considering that the device is still an experimental prototype. Even larger spreads of ratings were obtained when the subjects were asked about potential uses of the device. Nevertheless, on average they were quite confident that the device could have additional applications (e.g., teaching shapes). The interest in having the device spans all possible values (between 1 and 10), with median equal to 6. All subjects agreed that the device, as it is, has some limitations: the interest in getting a cheap, suitably engineered version of the device was much greater.

Subjects, after the experiment, provided some free comments. One subject said that he did not appreciate speech synthesis and supported any attempts to provide information in tactile form; another subject valued the flexibility of the device, given the possibility to provide three different modalities for representing braille letters, and also shapes; one subject underlined that the device, being portable, may contribute to improve the autonomy of blind people; many subjects suggested to enlarge the matrix of pins for tactile graphics applications; some subjects suggested to adapt our device to work similarly to Optacon (Linvill and Bliss, 1966), in order to allow accessing non-textual information. The latter indication could be simply addressed in future, as the available prototype already has a camera on the bottom side, from which images could be taken and reproduced in real-time by the tactile display (as mentioned in section 1).

## 4. Discussion

### 4.1. Related work

From the technological point of view, there are many approaches to provide tactile information, which can be categorized into thermal, electrical, mechanical actuators (for a complete review see Vidal-Verdu and Hafez, 2007 or Chouvardas et al., 2008). Thermal displays are based on actuators able to heat the skin locally, usually through Peltier cells (Hafez, 2007). They do not have a spatial resolution suited for graphical or textual applications (Vidal-Verdu and Hafez, 2007), probably due to the difficulties of making two adjacent actuators not to interfere with each other. Electrical stimulation has been studied as an alternative to mechanical stimulation (Kaczmarek et al., 1991). It offers the great advantage of not having moving parts and thus allowing more compact and lightweight solutions; on the opposite side, there is a great variety in users' perceptions and acceptance, thus making almost impossible to design a general solution (Vidal-Verdu and Hafez, 2007). Recent advances with a similar approach do not make use of electrical current passing through the user's skin, but still are not suited for braille applications (Xu et al., 2011). Mechanical solutions are the most investigated; usually they have piezoelectric, SMA (Shape Memory Alloy) or servomotor actuators (Vidal-Verdu and Hafez, 2007), but the first approach is the most used because of the good resulting trade off between spatial resolution, refresh rate and provided force (SMA and servomotor devices are able to apply a high force but they have a poor spatial resolution). Piezoelectric technology is also the most widespread among braille bars and similar devices. Emerging technologies, such as in the broad field of smart materials, are becoming a viable alternative for tactile applications, even if, due to their actual size, they can not be used yet in a real standard braille aid (Carpi et al., 2012).

Another important classification of different technical approaches is between static refreshable and dynamic displays (Vidal-Verdu and Hafez, 2007): the first ones aim to reproduce a large portion of graphics (or text) and let the user explore the whole content, the second ones provide a small portion of information (for example just one character at a time) and the content is dynamically refreshed according to the user's pace in reading or understanding the supplied information.

One of the first dynamic tactile displays was the Optacon (Linvill and Bliss, 1966; Bliss, 1969; Bliss et al., 1970): it had a rectangular matrix of 24 × 6 pins, spaced horizontally by 100mil and vertically by 50mil, moved by piezoelectric reeds with a cantilever system; the stimulation was by means of vibrations. It became very popular, since it was the first portable reader device to be produced and marketed. It did not support braille, as the acquired images of the glyphs were simply transposed onto the tactile display, not recognized and then translated into braille. Nevertheless, it enabled blind people to read printed text. It was a successful device, with the only drawback (regarding the tactile display) of stressing too much the fingertip (due to the adaptation phenomenon explained in section 1), thus making long usage difficult (Efron, 1977). Moreover, both hands were involved, one to scan the printed page, and the other held on the tactile transducer. To our knowledge, this device is not marketed anymore.

Using the same vibrotactile approach, the VITAL3 tactile display (Benali-Khoudja et al., 2007) offers a 8 × 8 matrix of actuators moved by microcoils directly printed on a PCB; micro-magnets are placed beneath a flexible membrane. In the third version of the prototype, the spacing between actuators is 3mm and each magnet has a diameter of 1.5mm. Thus, it should be a valuable option for representing braille (Dixon, 2010), but not for graphical applications (due to the high spacing between pins).

Regarding commercially available devices, two interesting solutions are the braille cell D2 [manufactured by Metec AG (Metec, 2012), which is the result of a research project aiming to develop a complete tactile graphical display (Völkel et al., 2008)] and the KGS SC5 graphic cell (KGS, 2012). They have a pin spacing of 2.5mm and 3.0mm, respectively, making them more similar to standard braille cells re-engineered to work as graphic cells, rather than devices designed for a “fine-grained” tactile stimulation.

### 4.2. Proposed solution

This paper introduces an innovative tactile display, able to provide tactile information through a high density matrix of pins. Analogously to most graphical tactile displays (Vidal-Verdu and Hafez, 2007), we have chosen a mechanical stimulation, based on piezoelectric actuators (this choice should reduce the cost barrier to make it an industrial product). In contrast with most of other solutions, we chose an indentation stimulation instead of a vibrotactile one, to avoid early adaptation (Efron, 1977; Levesque et al., 2007). However, it is interesting to note that having a refresh time of 10ms potentially makes it suitable for vibrotactile applications. Regarding the provided force, even if it is not as high as other mechanical devices (most notably those based on SMA), it is still effective for our purposes (Hale and Stanney, 2004), as shown by the results. The high resolution (pin diameter 0.8mm, gap between pins 0.7mm, scoring in the high range among the alternatives Vidal-Verdu and Hafez, 2007), near to the two-point limen for the fingertip, makes it a valuable option for mixed braille and graphical tactile applications; here we just focused on braille applications. A dual-use device would enable the user to switch easily between a *braille mode* and a *graphic mode*, depending on the contingent needs (and it is of course frequent in everyday life that textual and graphic information coexist in the same printed source). Finally, by including an MCU and an FPGA on-board, it is possible to customize the communication protocol and integrate high-level functionalities directly on the device.

An experimental protocol was designed to verify the performance of blind people in decoding tactile information encoded in braille. Different speeds and modalities of presentation of the characters were proposed.

**Figure A1:**
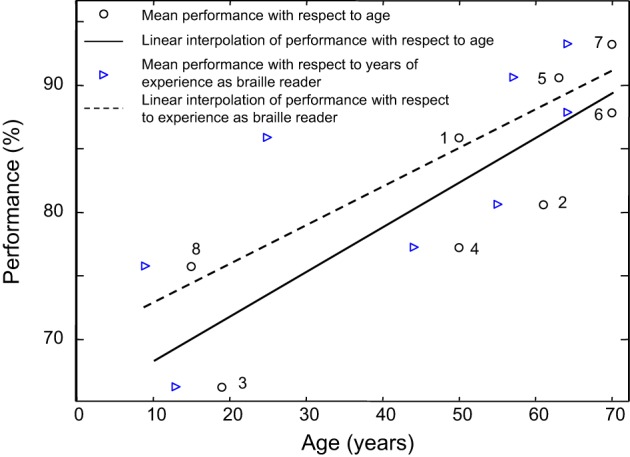
**Correlation between age or years of practice in braille reading (i.e., years since the subject started to study the braille code) and the mean performance of each of the eight subjects (each participant's performance is marked in accordance with Table 1)**.

**Figure A2:**
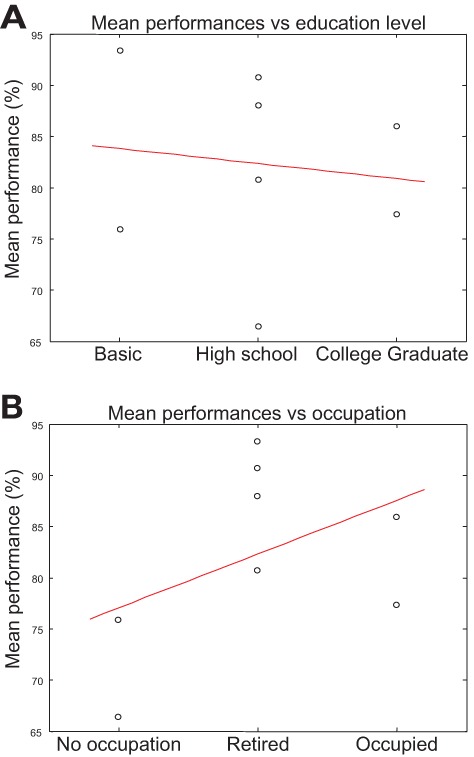
**Correlation between (A) education level or (B) occupation and the mean performance of each of the eight subjects**.

Many outcomes of the experiments are in line with our expectations. For example, as shown in Figure A1 in Appendix A, average performance of the subjects increased significantly as the experience of the braille reader increased (such an experience was measured in terms of the age or the years of practice in reading braille). Moreover, the preferred rate for a comfortable use of our device increased as the velocity in reading standard braille was higher, suggesting that the more the user is self confident with braille, the faster she/he can read with our device (even if this correlation was not statistically significant, probably due to the low number of participants involved in the experiments). On the other hand, performance was not statistically correlated with education and occupation. This result could be due to the high performance of young participants, who were keen of new technologies, but they didn't finish their studies and did not have an occupation (see Figure A2).

One of the most important properties of a new device is the appreciation by the user. Sometimes, blind people trying innovative devices reported that reading was increasingly difficult over time, due to either tactile adaptation or mental fatigue (Efron, 1977; Levesque et al., 2007). Our device, on the other hand, was well tolerated by the subjects, who performed experiments about 1 h long. It was easy to learn to use the device (5 min of introduction were enough). Moreover, using the device did not demand great concentration, as reported by subjects. Additionally, reading performance was statistically increasing over time: indeed, tests with different difficulty on recognizing words were performed in random order among different subjects; the recognition rate improved on average when the tests were considered in the order in which they were performed (Figure 9B). This is an objective indication that the subjects did not experience stress or fatigue, and training was quick and effective without assistance, on a short timescale (thereby suggesting room for further improvement).

Performance improved as the information content increased (Figure 9C), and this variation was statistically significant, despite the small sample. It was also expected that the performance could be higher when the rate of presentation of the characters was low (Figure 9D). Nevertheless, it is worth noticing that the performances are still acceptable (median of average subject performance larger than 80%) even using a speed 33% higher than the velocity considered as optimal by the subjects.

## 5. Conclusion and future perspectives

We have discussed the design, implementation and test of a dynamic tactile display for reconfigurable braille, featuring a high resolution tactile stimulation area allowing for customization of the braille layout, as well as the timing of the braille rendering, on a personal basis, thereby offering a flexible solution to match user's preferences and skills.

Even though the tests performed were encouraging, there is ample room for improvement, and the demonstrated device should be considered as a prototype for future engineering. Margins for improvement include the physical dimension (the device proved to be effectively usable, even with no previous training, but of course its usability would benefit from smaller size) and full implementation of Bluetooth communication.

Moreover, our device could be adapted to different usages from the one proposed to the subjects of our tests. For instance, using it for ebook reading would be an interesting option, which can be conceived in at least two ways: (1) the simplest would be just streaming the character content of the e-book to the device; this could be done in many ways, but would have the drawback of not allowing the active approach the blind user is used to; (2) a more sophisticated option would imply equipping the device with a displacement sensor: at each time, a software running on the PC hosting the ebook would identify the characters to be streamed for tactile transduction based on the “virtual exploration” performed by the user.

A further major improvement, which as already mentioned we believe is within reach, is the real-time translation of printed text into tactile dynamic braille patterns. Indeed, taking advantage of the camera mounted on the side opposite to the pin matrix in our prototype, and thanks to its easy reconfigurability, encouraging tests have been preformed, in which the device slides across the printed page, a custom OCR algorithm provides real-time character recognition, from which a tactile braille pattern is generated (Motto Ros, 2009). The performance of such a system was satisfactory, but further work is needed in order to obtain a portable all-in-one solution. Finally, the density of pins in our prototype, and the flexibility of firmware-level control, makes it feasible to provide—with the same device—the option of tactile rendering of graphic information; tests have been performed also in this direction (Mesin et al., 2010; Motto Ros and Pasero, 2011). Such a dual-mode braille-graphic device would constitute, we believe, a major step forward with respect to the state of the art.

### Conflict of interest statement

The authors declare that the research was conducted in the absence of any commercial or financial relationships that could be construed as a potential conflict of interest.

## Footnotes

^1^For reproducing braille text, the information carried by each tactile pin is binary, so we will not consider how to encode more data onto a single pin.

^2^It is noteworthy that this limit is not an issue in braille applications, and in any case it could be easily overcome by increasing the transfer speed, it is not a mechanical limit given by the actuators.

## A Appendix

### A.1 Additional data and experimental results

Table A1 reports participants' personal data. We assumed that there could be a relation between average performance in using our device and years of experience as a braille reader, or education level. Figures A1, A2 report measurements sensitive to such putative relations. Figure A1 shows the correlation between the mean performance (obtained averaging across all tests done by each subject) and age or years of braille practice. Correlation between age and average performance was quantified by a positive Spearman coefficient (*R* = 0.89), which was highly significant (*p* = 0.005). Correlation between the years of braille practice and the average performance was again quantified by a positive Spearman coefficient (*R* = 0.86), with statistical significance (*p* = 0.009). It is noteworthy that a significant result was obtained, for such a low number of subjects. However, caution is recommended before extending this preliminary indication to the whole population of blind people.

**Table A1:** **Participants' personal data: subject identifier, age, education level (B: basic; H: high school graduate; G: college graduate), occupational status (Y/R/N means occupied/retired/not occupied), age of blindness (years since the subject is blind), preferred character presentation time, reading rate (on standard braille), braille configuration, average qualitative indication**.

**Subject**	**Age**	**Education**	**Occupational status**	**Age of blindness**	**Character persistence (ms)**	**Reading rate (chars/_s_)**	**Braille configuration**	**Qualitative indication**
1	50	G	Y	25	500	1.7	3	5.75
2	61	H	R	61	700	3.4	1	6.25
3	19	H	N	19	750	4	1	9
4	50	G	Y	50	450	7.7	1	7.17
5	63	H	R	63	500	5	1	4.25
6	70	H	R	70	600	7.5	2	4.91
7	70	B	R	70	600	8	1	4.91
8	15	B	N	2	450	7	1	7.17

Figure A2 shows the relation between education level or occupation and mean performance. Linear fits were statistically poor results for the relation between performance and education level (Spearman coefficient *R* = −0.15, not significant, *p* = 0.77) and for the relation between performance and occupation (Spearman coefficient *R* = 0.38, not significant, *p* = 0.39).
